# Highlights of American Society of Hematology 2024—lymphoma

**DOI:** 10.1093/oncolo/oyaf166

**Published:** 2025-06-30

**Authors:** Alexander D Sanjurjo, Barbara Pro, Hua-Jay J Cherng

**Affiliations:** Columbia University Vagelos College of Physicians & Surgeons, New York, NY, USA; Division of Hematology & Oncology, Columbia University Irving Medical Center, New York, NY, USA; Division of Hematology & Oncology, Columbia University Irving Medical Center, New York, NY, USA

## Introduction

Here, we present key highlights from the 66th Annual Meeting of the American Society of Hematology (ASH), held from December 7–10, 2024, in San Diego. With over 30 000 attendees from more than 100 countries, the conference featured numerous impactful studies. This article highlights a curated selection of studies with the potential to change clinical practice in lymphoma.

## Large B-cell lymphoma

Many trials at ASH 2024 involving newly diagnosed large B-cell lymphoma (LBCL) explored adding novel agents to standard immunochemotherapy backbones.

To address the high rate of relapse in high-risk LBCL the COALITION trial (**Abstract 582**) was a phase 2, multi-center study in Australia assessing the addition of glofitamab, a CD20xCD3 bispecific antibody (BsAb) approved for relapsed/refractory (R/R) LBCL, to R-CHOP (rituximab, cyclophosphamide, doxorubicin, vincristine, and prednisone) and pola-R-CHP (polatuzumab vedotin, rituximab, cyclophosphamide, doxorubicin, and prednisone) in younger patients with high-risk LBCL.^1^ After receiving one cycle of R-CHOP, 80 patients with untreated LBCL with at least 1 high-risk feature (International Prognostic Index ≥ 3, National Comprehensive Cancer Network-International Prognostic Index ≥ 4, or high-grade LBCL with MYC and BCL2 and/or BCL6 rearrangements) were randomized to 5 cycles of glofitamab with either R-CHOP or pola-R-CHP followed by 2 cycles of glofitamab. The median follow-up was 20.7 months. At the end of induction, complete response (CR)/partial response rates were 70%/29% in the glofitamab R-CHOP arm, and 80%/18% in the glofitamab pola-R-CHP arm. At 12 months, overall survival (OS)/progression-free survival (PFS) were 88%/96% (glofitamab R-CHOP) and 95%/97% (glofitamab pola-R-CHP). Further follow-up is needed, and larger randomized studies, such as the phase 3 study SKYGLO (NCT06047080), are underway.^2^

As BsAb continues to show promise in R/R LBCL, recent real-world studies presented at ASH 2024 further defined their role.

The CD20xCD3 BsAb glofitamab and epcoritamab were recently approved for R/R LBCL. However, most data come from clinical trials.^3,4^ To assess real-world outcomes, the REALBiTE study (**Abstract 111**), a multi-center retrospective cohort study across 19 US centers, investigated outcomes of 209 patients with R/R LBCL receiving either glofitamab or epcoritamab from 2023-2024.^5^ Most patients (60%) received prior chimeric antigen receptor T-cell (CAR T) therapy. The overall response rate (ORR) was 50.6% and the CR rate was 23.8%. With a median follow-up of 5 months, the median PFS was 2.7 months, and the median OS was 7.2 months. Those without detectable CD20 expression on IHC or flow cytometry prior to BsAb therapy experienced significantly worse outcomes compared to those with detectable CD20, with PFS 1.1 months vs. 3.4 months (*P* < .001) and OS 1.3 vs. 13 months (*P* < .001).

**Abstract 114** ^6^ was a retrospective, multi-center analysis of 85 patients with R/R LBCL who received BsAb after CAR-T failure. Among them, 88% were treated with glofitamab and 10.6% with epcoritamab. Although most patients (60%) received BsAb treatment immediately after CAR T, 40% received BsAb as a later salvage therapy. Overall, 42% of patients responded to BsAb antibody treatment: 23% achieved a CR and 19% had a partial response. On further subgroup analysis, patients were divided into 3 groups based on time of CAR-T failure: early (within first 3 months), intermediate (4-6 months), and late (>6 months). BsAb were found to be more efficacious in patients with later relapse after CAR T treatment, as there were differences noted in ORR (*P* = .027), CR rate (*P* = .006), median PFS (*P* = .002), and median OS (*P* = .0002).

Together, these studies demonstrate that BsAb underperforms in real-world settings versus clinical trials, particularly for those with early relapse after CAR T. This may be due to shared factors affecting T-cell redirecting therapy efficacy such as T-cell exhaustion, immune evasion, or development of an “immune cold” microenvironment.

## Follicular lymphoma

Most patients with follicular lymphoma (FL) eventually relapse after treatment. While non-cytotoxic novel therapies are preferred in the R/R setting, improving the durability of response remains a key challenge. Following the success of a phase 2 trial of loncastuximab teserine, an anti-CD19 antibody-drug conjugate,^7^ researchers are now investigating new applications for anti-CD19 therapies in R/R FL.

**Late-Breaking Abstract 1** (**LBA-1**), the phase 3 inMIND study,^8^ evaluated the use of tafasitamab, a humanized CD19 monoclonal antibody approved for R/R DLBCL,^9^ in combination with lenalidomide and rituximab for patients with R/R FL. In total, 548 patients with R/R FL were randomized 1:1 to either tafasitamab or placebo, with all patients receiving lenalidomide and rituximab. With a median follow-up of 14.1 months, a significant improvement in median PFS was observed: 22.4 months for the tafasitamab arm compared to 13.9 months in the placebo arm (HR 0.43, *P* < .0001). This benefit was consistent throughout high-risk subgroups of patients including those with progression of disease within 24 months (POD24), patients refractory to prior anti-CD20 therapy, and those receiving multiple prior lines of treatment. Notably, the PFS curve for tafasitamab showed sustained separation from the placebo but did not demonstrate a plateau, suggesting a continual risk of relapse. Although longer follow-up for OS data is needed, this was the first study to validate the combination of 2 monoclonal antibodies in the treatment of lymphoma.

Another study targeting CD19, **Abstract 341**^10^ is an ongoing phase 1 study evaluating dose escalation of AZD0486, a humanized CD19xCD3 bispecific T-cell engager, in R/R FL and other B-cell NHL. With a median follow-up of 8.2 months, 47 patients with FL R/R to ≥2 previous lines of therapy received varying doses of AZD0486, with those receiving ≥2.4 mg demonstrating a 95% ORR and a CR rate of 85%. This included patients with CD20-negative disease and heavily pretreated patients (including with CD20 BsAb and CD19 CAR T). High rates of undetectable measurable residual disease (MRD), assessed with circulating tumor DNA (ctDNA) sequencing, were seen in patients receiving a dose ≥2.4 mg with CR (92% within 12 weeks). While follow-up remains short, the PFS curve, particularly at the 7.2 mg dose, has remained relatively flat beyond 12 months, raising the possibility of durable remissions in a subset of patients. Only grade 1 cytokine release syndrome and immune effector cell-associated neurotoxicity syndrome (ICANS) were observed, with no treatment-related discontinuations. Overall, it appears that AZD0486 can be safely administered and leads to high response rates in R/R FL.

When considering the breadth of CD19 targeting therapies described, such as CAR T, loncastuximab teserine, tafasitamab, and AZD0486, it appears that CD19 has become a target of high significance in the R/R setting.

## Mantle cell lymphoma

Although patients with mantle cell lymphoma (MCL) have traditionally received autologous stem cell transplantation (ASCT) consolidation following CR to intensive induction immunochemotherapy,^11^ the phase 3 ECOG-ACRIN EA4151 trial (**LBA-6**)^12^ examined the efficacy of ASCT for MCL patients achieving deep first molecular remission, regardless of induction regimen. Among 650 patients in CR after first-line therapy, those with undetectable MRD (uMRD) measured by clonoSEQ next-generation sequencing were randomized 1:1 to 3 years of maintenance rituximab (MR) plus ASCT or 3 years of MR alone. Those with MRD positive or MRD indeterminate CR received ASCT plus MR. At 2.7 years median follow-up, there was no difference in 3-year PFS for all uMRD patients receiving MR + ASCT vs. MR alone (HR 1.05, *P* = .79). In an exploratory analysis, MRD-positive patients who converted to uMRD after ASCT had a 3-year OS and PFS of 100%. In contrast, those who remained MRD-positive post-ASCT had a 3-year OS of 63.6% and PFS of 48.8%. These findings suggest that ASCT may not provide additional benefits for patients experiencing MRD negativity after induction therapy and could possibly be omitted in this population. In contrast to the TRIANGLE trial,^13^ only 7.2% of patients in this trial received a Bruton tyrosine kinase (BTK) inhibitor as part of frontline therapy, limiting generalizability to modern treatment paradigms.

However, recent data from the TRIANGLE trial suggested that ASCT may not add benefit to modern BTK inhibitor-containing induction regimens either. At ASH, the authors presented extended follow-up from the trial in **Abstract 240**.^14^ The trial randomized previously untreated stage II-IV MCL patients 1:1:1 to one of three arms: (1) standard treatment with R-CHOP or rituximab, cisplatin, cytosine arabinoside, and dexamethasone (R-DHAP), each followed by ASCT, (2) standard treatment with ibrutinib, and (3) standard treatment with ibrutinib but without ASCT. It confirmed 2 key findings: First, it reaffirmed that an ibrutinib-containing regimen without ASCT showed superiority over standard treatment in terms of failure-free survival and OS (*P* = .0102 and *P* = .0041, respectively). Second, and the key finding from this abstract, adding ASCT to an ibrutinib-containing regimen did not improve failure-free survival (*P* = .28) and instead increased toxicity. These results reinforce the lack of benefit of ASCT in younger patients receiving BTK inhibitors as part of induction.

Overall, in the era of BTK inhibitors and with the growing role of MRD testing, consolidation with ASCT appears to be becoming less relevant in MCL. Patients achieving MRD negativity after induction, including with BTK inhibitors, may represent a low-risk group where omitting ASCT could be considered.

## Hodgkin lymphoma

This year’s ASH meeting featured several important updates in the management of Hodgkin lymphoma (HL), highlighting a continued shift toward response-adapted strategies and the integration of novel agents into both frontline and R/R settings.

The RATHL study in 2016 showed that for patients with advanced-stage HL, bleomycin can be omitted from subsequent cycles if the PET scan after ABVD × 2 cycles (PET2) is negative.^15^**Abstract 461**^16^ was a single-center study in Canada that investigated whether the same paradigm could be applied to limited stage HL, defined as stage IA, IB, or IIA, and non-bulky disease. Patients were diagnosed between 2011 and 2022, allowing for an era-to-era comparison of outcomes in PET2-negative patients treated with ABVD ×4 versus ABVD/AVD ×2. The majority (85%) were treated per the prevailing standard of care at the time. Five-year PFS (92.3% vs. 94.9%, *P* = .504) and 5-year OS rates (98.4% vs. 100%, *P* = .220) were similar between the ABVD and AVD eras. Pulmonary toxicity, determined by the treating physician, was lower with AVD, occurring in only 3% of patients compared to 16% with ABVD. These findings support the omission of bleomycin for cycles 3 and 4 following a negative PET2 scan in limited-stage HL, though a larger sample size is needed to detect significant differences in pulmonary toxicity and further follow-up is needed in the later era AVD cohort.

Since the approval of brentuximab vedotin in combination with doxorubicin, vinblastine, and dacarbazine (AVD) for advanced HL, researchers have explored incorporating additional novel agents into this regimen. The phase 2 SGN35-027 Part C study previously demonstrated promising efficacy and a favorable safety profile for adding nivolumab to a combination of brentuximab vedotin, doxorubicin, and dacarbazine (AN + AD), omitting vinblastine based on prior evidence suggesting preserved efficacy in early-stage, non-bulky HL.^17^**Abstract 460**^18^ presented extended follow-up, increasing the median follow-up period from 16.5 months to 30.7 months. The study included 154 patients in Germany diagnosed with early-stage (stage I or II), non-bulky HL receiving 4 cycles of AN + AD. ORR at the end of treatment was 96% and CR rate was 92%, with a 36-month PFS rate of 95%. In terms of safety, grade ≥ 3 treatment-related adverse events occurred in 34% of patients (mainly neutropenia and AST/ALT increase), while grade ≥ 3 immune-mediated adverse events occurred in 8% of patients (mainly ALT increase and rash). These results support AN + AD as a durable and well-tolerated frontline option in patients with early-stage non-bulky HL.

For patients with R/R HL, the standard approach is second-line therapy to treat CR followed by ASCT. A 2021 phase 2 study evaluating second-line therapy with pembrolizumab, gemcitabine, vinorelbine, and liposomal doxorubicin (P-GVD) followed by ASCT showed 95% achieved CR and 96% PFS at 30 months.^19^ In part II (**Abstract 569**), the authors explored whether those who achieved CR after P-GVD could avoid ASCT.^20^ Among 40 enrolled patients, 36 achieved CR following P-GVD and 24 proceeded to receive 13 cycles of pembrolizumab maintenance with no ASCT. At a median follow-up of 23.4 months, 2-year PFS was 51%, with 9 of the 10 that progressed successfully salvaged with third-line therapy and ASCT. While a randomized study is ongoing, this shows that pembrolizumab maintenance may not be sufficient and ASCT remains the best curative option.

## T-cell lymphoma

Treatment outcomes for R/R T-cell lymphomas remain poor, with few therapies offering durable benefits. While most investigational therapies have yet to shift clinical practice, some emerging combinations show promise in early-phase trials. This year, 2 notable abstracts highlighted novel combination regimens with encouraging efficacy in R/R disease.

**Abstract 463** ^21^ studied duvelisib (PI3K-γδ inhibitor) plus ruxolitinib (JAK1/2 inhibitor) as a dual-targeted therapy in a multi-center phase 1 trial. It enrolled 49 patients with R/R peripheral T-cell lymphoma (PTCL), cutaneous T-cell lymphoma (CTCL), or untreated T-prolymphocytic leukemia (T-PLL). Part I evaluated the maximum tolerated dose while part II compared efficacy between patients with and without genetic or immunohistochemical JAK/STAT activation. ORR was 41% overall, with a 24% CR rate. Response was better in patients with JAK/STAT activation, as they had an ORR of 52% and CR rate of 29%, compared to 14% ORR and CR rate in those without (*P* = .023). When stratified by subtype, T-follicular helper (TFH) lymphomas, and T-PLL responded best to the therapy (ORR 79% and 60%, respectively). Given that these two histologies represented 19 patients total, the further expansion for T-PLL and TFH lymphomas are being planned.

Lastly, though romidepsin, azacitidine, and lenalidomide have all shown single-agent activity against R/R PTCL, and lenalidomide and romidepsin have each shown improved CR rates when combined with azacitidine,^22,23^ a combination regimen of all 3 has not been tested due to concerns for toxicity. A phase 1 trial (**Abstract 984**) tested escalating doses of lenalidomide (5-20 mg) with fixed doses of romidepsin, azacitidine, and dexamethasone (RAdR) on 26 patients with R/R PTCL over 6 28-day cycles.^24^ The maximum tolerated dose of lenalidomide was 20 mg, with 23% discontinuing due to toxicity (mostly thrombocytopenia). In the 25 patients that received RAdR, ORR was 63% with a CR rate of 19%. With a median follow-up of 18.2 months, 1-year PFS and OS were 16.9% and 70.3%. The 9 patients receiving the maximum tolerated dose had a particularly high rate of response, with an ORR of 89% but no major differences in CR rate, PFS, or OS. These responses were active across different PTCL subtypes; therefore this 4-drug regimen may represent an effective therapeutic bridge to transplantation upon further studies.

## Conclusion

The 2024 ASH meeting highlighted several emerging trends that could reshape clinical practice in lymphoma. Across subtypes, novel targeted therapies and their combinations demonstrated promising efficacy and safety in both frontline and salvage settings. The growing adoption of response-adapted and de-escalation strategies is also redefining treatment paradigms, aiming to maintain outcomes while reducing toxicity. Together, these findings signal a shift toward more personalized, biomarker-driven approaches that optimize efficacy and minimize treatment burden.

## Data Availability

No new data were generated or analyzed in support of this research.
